# Metabolic Effects of Testosterone Replacement Therapy in Patients with Type 2 Diabetes Mellitus or Metabolic Syndrome: A Meta-Analysis

**DOI:** 10.1155/2020/4732021

**Published:** 2020-09-30

**Authors:** Shu-ying Li, Ya-ling Zhao, Yu-fan Yang, Xi Wang, Min Nie, Xue-yan Wu, Jiang-feng Mao

**Affiliations:** Peking Union Medical College, Chinese Academy of Medical Sciences, Peking Union Medical College Hospital, Division of Endocrinology, Beijing 100730, China

## Abstract

**Background:**

Testosterone replacement therapy (TRT) is commonly used for the treatment of hypogonadism in men, which is often associated with type 2 diabetes mellitus (T2DM) and metabolic syndrome (Mets). Recent compiling evidence shows that TRT has beneficial metabolic effects on these patients.

**Objective:**

A meta-analysis has been conducted to evaluate the effects of TRT on cardiovascular metabolic factors.

**Methods:**

We conducted a systemic search on PubMed, Embase, Cochrane Library, Wanfang, and CNKI and selected randomized controlled trials (RCTs) to include. The efficacy of TRT on glycemia, insulin sensitivity, lipid profile, and body weight was meta-analyzed by Review Manager.

**Results:**

A total of 18 RCTs, containing 1415 patients (767 in TRT and 648 in control), were enrolled for the meta-analysis. The results showed that TRT could reduce HbA1c (MD = −0.67, 95% CI −1.35, −0.19, and *P*=0.006) and improve HOMA-IR (homeostatic model assessment of insulin resistance) (SMD = −1.94, 95% CI −2.65, −1.23, and *P* < 0.0001). TRT could also decrease low-density lipoprotein (SMD = −0.50, 95% CI −0.82, −0.90, and *P*=0.002) and triglycerides (MD = −0.64, 95% CI −0.91, −0.36, and *P* < 0.0001). In addition, TRT could reduce body weight by 3.91 kg (MD = −3.91, 95% CI −4.14, −3.69, and *P* < 0.00001) and waist circumference by 2.8 cm (MD −2.80, 95% CI −4.38, −1.21 and *P*=0.0005). Erectile dysfunction (measured by IIEF-5) did not improve, while aging-related symptoms (measured by AMS scores) significantly improved.

**Conclusions:**

TRT improves glycemic control, insulin sensitivity, and lipid parameters in hypogonadism patients with T2DM and MetS, partially through reducing central obesity.

## 1. Introduction

Male hypogonadism is defined as insufficient testosterone due to variable pathology in any part of the hypothalamus-pituitary-testes axis [1]. It presents with primary symptoms of decreased libido, erectile dysfunction, and infertility. The incidence of male hypogonadism increases with age [2], with the prevalence in men aged 30–79 years at 3.1–7.0%, which increases markedly to 18.4% among men over 70 years [3]. Hypogonadism has a profound negative impact on both the physical health and the life satisfaction for middle-aged and elderly men.

Previous studies have reported a complex relationship between low testosterone levels and deteriorating metabolic status, such as hyperglycemia, obesity, and poor lipid profile [3]. The incidence of androgen deficiency in male patients with metabolic syndrome (MetS) and type 2 diabetes mellitus (T2DM) is significantly higher than that in the normal population [4]. Studies have found that the prevalence of hypogonadism in T2DM is 46% (*n* = 333) [5] in Poland and 33.1% (*n* = 112) in China [6]. MetS is a group of clinical syndromes consisting of obesity, hypertension, hyperglycemia, dyslipidemia, and other metabolic disorders. It is a major risk factor for T2DM and cardiovascular diseases. Considering the close interlinked relationship between diabetes, obesity, and hypogonadism [6, 7], we believe that further investigation in the causality among these factors is worthwhile.

Testosterone replacement therapy (TRT) is the primary treatment for male hypogonadism [8]. It has been confirmed that TRT can improve the symptoms of hypogonadism [1, 8]; however, the metabolic effects of this treatment on male hypogonadism remain controversial. Evidence supporting TRT improvement of glucose control, lipid profile, and weight control is still insufficient. For this reason, screening for testosterone deficiency and TRT for men with T2DM and MetS is not routinely recommended in some countries [9]. In recent years, compiling evidence consistently shows that TRT can improve metabolic factors, a compelling issue that leads us to conduct this meta-analysis.

## 2. Materials and Methods

Following the reporting recommendations made by the PRISMA (Preferred Reporting Items for Systemic Reviews and Meta-Analyses) statement, we met all 27 items stated therein in our study.

## 3. Objectives

### 3.1. Inclusion Criteria

The inclusion criteria are fully published, randomized, and controlled clinical trials; aiming to evaluate the metabolic effects of TRT on patients with MetS and/or T2DM; language in English or Chinese.

### 3.2. Exclusion Criteria

The exclusion criteria are (1) case reports and reviews, (2) language in non-Chinese or non-English, (3) republished articles, (4) literature studies in which data were missing or unavailable, and (5) literature studies that did not provide standard deviation or quartile spacing.

Groups: the TRT group was defined as patients treated with testosterone supplementation. The control group was defined as a blank or receiving placebo.

### 3.3. Search Strategy

**Figure 1:**
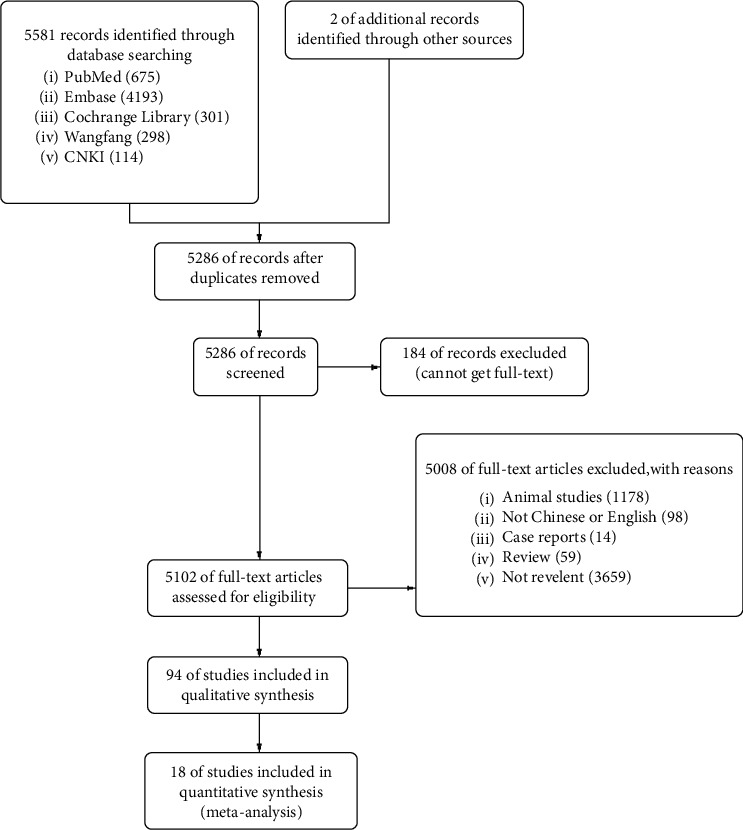
Flow chart summarizing the screening strategy for studies included for meta-analysis.

The search strategy was designed by an expert on public health (Figure 1). Databases, including PubMed, Embase, Cochrane Library, CNKI, and Wanfang, were searched in order to identify qualified trials published up to Dec 2019. Potentially eligible studies on the reference lists were searched by hand. Meta-analysis was conducted by two independent reviewers (Li and Zhao). Discrepancies were resolved by discussion or submitted to a third party.

### 3.4. Data Extraction

Two investigators, Li and Zhao, extracted the relevant data independently using a standardized form. The data included demographic information, diagnosis of T2DM and/or MetS, number of participants, baseline testosterone levels, therapeutic regimen, and treatment duration. Differing opinions were resolved by consulting a third party.

### 3.5. Quality Assessment

**Figure 2:**
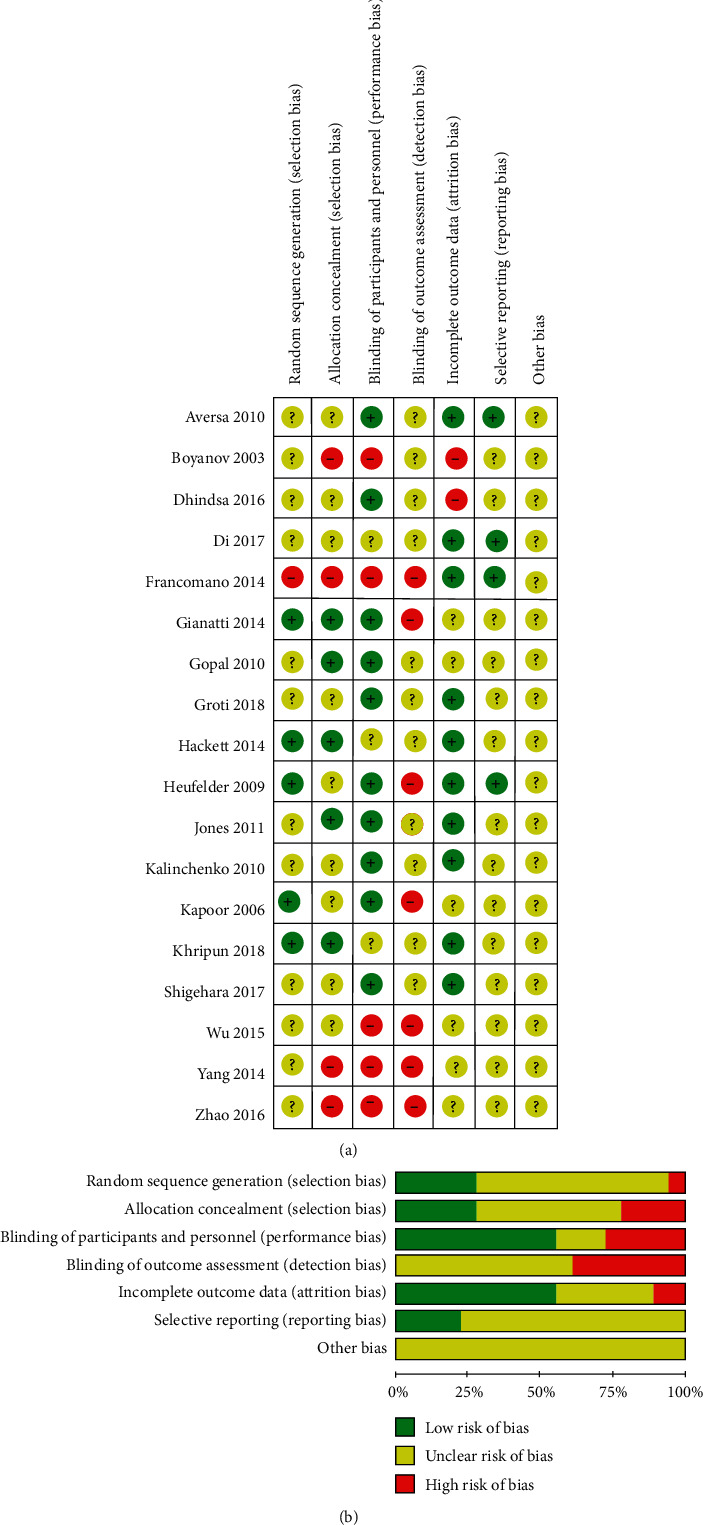
Risk bias assessment.

Methodological quality evaluation was performed using a quality evaluation tool recommended by the Cochrane Reviewers' Handbook (Figure 2).

### 3.6. Primary Outcome

The primary outcomes following TRT were improvement of glycemia, lipid parameters (HDLc, LDLc, and triglyceride), body weight, and waist circumference.

### 3.7. Secondary Outcome

The secondary outcome upon TRT was improvement of sexual function, as assessed by the International Index of Erectile Function-5 (IIEF-5) scores, as well as symptoms of senescence, as assessed by the Aging Males Symptoms (AMS) scores. In addition, we also evaluated the possible adverse effects of TRT, including blood pressure, prostate-specific antigen (PSA), hemoglobin, and hematocrit.

### 3.8. Data Synthesis and Statistical Analysis

The two investigators, Li and Zhao, independently analyzed the extracted data. Review Manager (RevMan5.3) software was used to generate the meta-analysis of the clinical efficacy from various studies. The heterogeneity of the included literature studies was firstly analyzed by the *I*^2^ test. The fixed-effect model was adopted if *I*^2^ was <50%. Otherwise, a random-effect model would be adopted. A funnel plot was used to analyze publication bias. In clinical studies, if a standard deviation (SD) was not provided, it was calculated using the following formula: the required correlation coefficient Corr was directly obtained or obtained through the included studies [10–12]:

(1) $$\begin{array}{c} {\text{SD}}_{\text{change}}^{2}={\text{SD}}_{\text{baseline}}^{2}+{\text{SD}}_{\text{final}}^{2}-2\times \text{Corr}\times {\text{SD}}_{\text{baseline}}\times {\text{SD}}_{\text{final}}. \end{array}$$

### 3.9. Ethical Approval

This meta-analysis review does not require approval from an ethics committee.

## 4. Results

### 4.1. Study Identification and Descriptive Data Synthesis

The computer initially retrieved 5,581 literature articles. After removing duplicating articles, animal experimental studies, non-Chinese and non-English studies, reviews, case reports, and unrelated studies, we enrolled 18 [10–27] RCTs meeting the inclusive criteria (Figure 1). A total of 1415 participants, 767 in the TRT group and 648 in the control group, were eligible for data analysis. The characteristics of the included studies are shown in Table 1. The quality evaluation for the included studies is shown in Figure 2.

### 4.2. The Effects of TRT on Glucose Metabolism

#### 4.2.1. The Effects of TRT on HbA1c and Fasting Blood Glucose

**Figure 3:**
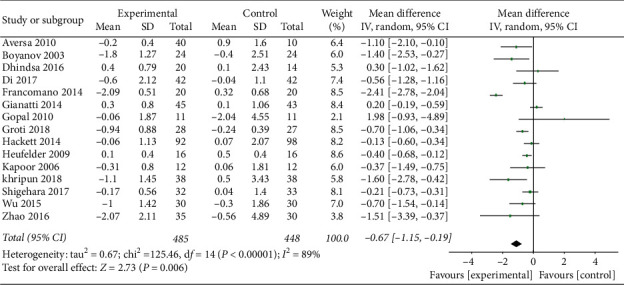
TRT results in increased HbA1c reduction compared to the control group.

A total of 15 studies were included to evaluate the effect of TRT on glycosylated hemoglobin (HbA1c) (Supplementary Table S1). The results of our analysis showed that TRT significantly reduced HbA1c by 0.67% (MD = −0.67, 95% CI −1.35, −0.19, and *P*=0.006) (Figure 3).

A subgroup analysis performed according to the duration of TRT identified that the study by Khripun et al. [15] was the source of heterogeneity. TRT would improve HbA1c by −0.58 (95% CI −1.08, −0.08), 0.06 (95% CI −0.26, 0.38), and −0.50 (95% CI −0.74, −0.24) in patients undergoing TRT for a duration of ≤6 months, 6–12 months, and ≥12 months, respectively (Supplementary Figure S1).

A subgroup analysis performed according to baseline HbA1c level found that TRT would improve HbA1c by −0.32 (95% CI −0.62, −0.03) and −0.76 (95% CI −1.07, −0.44) in patients with baseline HbA1c ≤8 and >8%, respectively (Supplementary Figure S2).

A total of 15 studies (676 patients in TRT and 588 patients in control) were enrolled in order to clarify the effect of TRT on fasting blood glucose (FBG). The findings revealed that TRT significantly changed FBG by −0.86 mmol/l (95% CI −1.15, −0.56, and *P* < 0.001) (Supplementary Figure S3).

We next conducted subgroup and sensitivity analysis according to the duration of TRT. The results indicated that the studies by Dhindsa et al. [17], Khripun et al. [15], Heufelder et al. [26], and Yang [23] were the sources of heterogeneity (Supplementary Figure S4).

#### 4.2.2. The Effect of TRT on Insulin Sensitivity (Fasting Insulin and HOMA-IR)

We evaluated the effect of TRT on fasting insulin (FINS) by enrolling 11 studies (473 patients in TRT and 367 patients in control). Our findings revealed that TRT significantly reduced FINS (SMD = −1.23, 95% CI −1.85, −0.62, and *P* < 0.0001) (Supplementary Figure S5).

**Figure 4:**
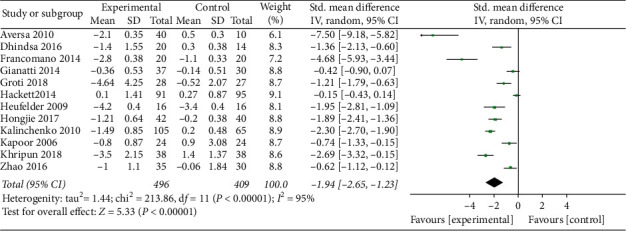
TRT significantly improves HOMA-IR.

We also investigated the effect of TRT on HOMA-IR by enrolling 12 studies (496 patients in TRT and 409 patients in control). The results indicated that TRT could significantly reduce HOMA-IR (SMD = −1.94, 95% CI −2.65, −1.03, and *P* < 0.0001) (Figure 4).

### 4.3. The Effects of TRT on Lipid Profile (TC, TG, LDL, and HDL)

To evaluate the effect of TRT on total cholesterol (TC), a total of 15 studies were included in the analysis, and the results of which showed a significant reduction in TC (SMD = −0.86, 95% CI −1.31, −0.40, and *P* < 0.0001) (Supplementary Figure S6).

**Figure 5:**
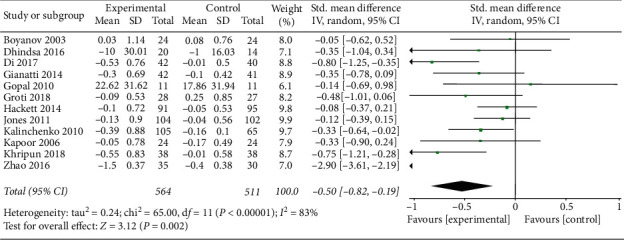
TRT can reduce LDLc.

Twelve studies (564 patients in TRT and 511 patients in control) were included to clarify the effect of TRT on LDLc. The data showed a significant reduction in LDLc (SMD = −0.50, 95% CI −0.82, −0.19, and *P*=0.002) (Figure 5).

A total of 17 studies (735 patients in TRT and 612 patients in control) were included in order to evaluate TRT's effect on triglyceride (TG) levels. Overall, we found that TRT significantly reduced TG (SMD = −0.64, 95% CI −0.91, −0.36, and *P* < 0.0001) (Supplementary Figure S7).

Next, we evaluated the effect of TRT on HDLc by including 15 studies (682 patients in TRT and 562 patients in control). Overall, the change of HDLc after TRT was nonsignificant (SMD = 0.18, 95% CI −0.12, 0.48, and *P*=0.79).

Changes in the lipid profile following TRT are summarized in Supplementary Table S2.

### 4.4. The Effects of TRT on Body Composition (Weight, BMI, and WC)

Seven studies (343 patients in TRT and 299 in control) were included for the evaluation of TRT's effect on body weight. We found that the inter-trial heterogeneity was not significant (Cochrane *Q*-test *P*=0.68, *I*^2^ = 0%), and so we adopted a fixed-effect model for combining the data. Testosterone therapy resulted in an average weight loss of 3.91 kg (MD = −3.91, 95% CI −4.14, −3.69, and *P* < 0.00001) (Supplementary Figure S8).

**Figure 6:**
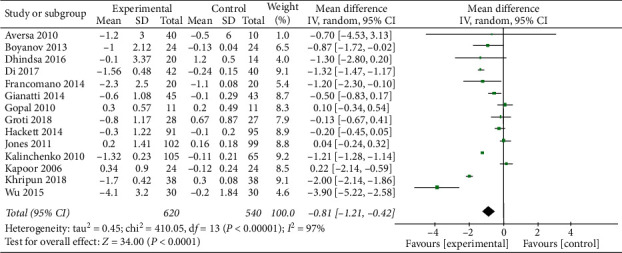
TRT can reduce BMI.

We also included a total of 14 studies (620 patients in TRT and 540 in control) to investigate the change in BMI after TRT. The results showed that TRT significantly reduced BMI (MD = −0.81, 95% CI −1.21, −0.42, and *P* < 0.0001) (Figure 6).

Additionally, we evaluated the change in waist circumstance after TRT by including 14 studies (656 patients in TRT and 539 patients in control). We found that TRT significantly reduced waist circumstance by 2.8 cm (MD = −2.8, 95% CI −4.38, −1.21, and *P*=0.0005) (Supplementary Figure S9).

The changes in weight, BMI, and waist circumstance are summarized in Supplementary Table S3.

### 4.5. The Change in Testosterone Levels after TRT

Overall, the total testosterone in serum increased (among the included 12 studies) following TST when compared to the controls (SMD = 1.92, 95% CI 4.61, 878, and *P* < 0.0001) (Supplementary Figure S10).

### 4.6. The Effects of TRT on Erectile Function and Aging-Related Symptoms

To evaluate the effect of TRT on the IIEF-5 score, a total of 5 studies (310 patients in TRT and 275 patients in control) were included. The findings indicated that TRT did not significantly improve the IIEF-5 score when compared to controls (SMD = 1.55, 95% CI −0.43, 3.54, and *P* < 0.0001, data not shown).

We included a total of 6 studies (362 patients in TRT and 325 patients in control) for the evaluation of TRT's effects on AMS scores. We found that testosterone therapy significantly reduces the AMS score (MD = −4.65, 95% CI −8.76, −0.54, and *P* < 0.0001) (Supplementary Figure S11), indicating that TRT improves aging-related symptoms.

### 4.7. The Safety of Testosterone Replacement Therapy

Changes in systolic blood pressure (SBP) following TRT were evaluated through the inclusion of 12 studies (465 patients in TRT and 393 controls). We found that TRT did not significantly influence SBP (MD = −0.31, 95% CI −0.89, 0.28, and *P*=0.91, data not shown).

Furthermore, changes in diastolic blood pressure (DBP) after TRT were evaluated through the inclusion of 13 studies (478 patients in TRT and 419 controls), which revealed no significant difference after TRT (MD = −0.67, 95% CI −3.21, 1.88, and *P*=0.61, data not shown).

We then evaluated the effect of TRT on PSA by examining 3 studies (122 patients in TRT and 91 in control). Because the inter-trial heterogeneity was not significant (Cochrane *Q*-test *P*=0.14, *I*^2^ = 0%), we adopted a fixed-effect model for combining the data. The results showed that TRT did not increase PSA (MD = −0.12, 95% CI −0.06, 0.30, and *P*=0.20 ) (Supplementary Figure S12).

Next, we analyzed the effect of TRT on hemoglobin and hematocrit by including 3 studies (125 patients in TRT and 93 controls). The data revealed an increase in the hemoglobin level of 1.49 g/dl (MD = 1.49, 95% CI 1.04, 1.93, and *P* < 0.00001) (Supplementary Figure S13), and an increase in the hematocrit (MD = 0.16, 95% CI −0.00, 0.33, and *P*=0.05) (Supplementary Figure S14).

Changes in the blood pressure, PSA, and hemoglobin after TRT are summarized in Supplementary Table S4.

### 4.8. Risk of Publication Bias

**Figure 7:**
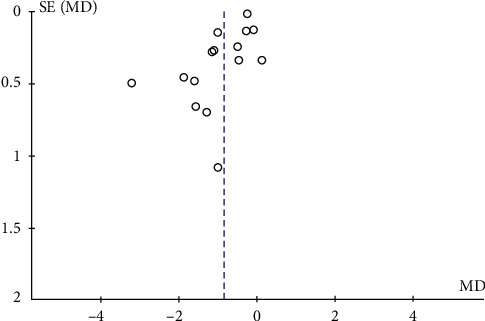
Publication bias was evaluated by the change of HbA1c after TRT.

Publication bias was evaluated by examining the change of HbA1c after TRT. The funnel plot showed that the distribution was symmetrical with slight publication bias (Figure 7).

## 5. Discussion

We employed rigorous inclusion criteria in this meta-analysis, ultimately including 18 studies. We then set out to systemically investigate the effects of TRT on HbA1c, LDLc, body weight, waist circumstance, blood pressure, and hemoglobin. Our analysis concluded that TRT had favorable metabolic effects on glycemia control, lipid profile, and weight loss.

Our findings indicated that testosterone supplementation could improve glycemia control. This is based on our observation that TRT reduced HbA1c by 0.67%, fasting blood glucose by 0.86 mmol/L, and fasting insulin and insulin resistance index (HOMA-IR) by 1.23. There was significant heterogeneity in the meta-analysis of HbA1c, and sensitivity analysis revealed that the heterogeneity was primarily derived from differences in treatment duration. Subgroup analysis according to treatment duration showed that HbA1c did not improve in patients who underwent TRT for 6∼12 months, possibly due to the small sample size. Another subgroup analysis, stratified by baseline HbA1C, showed that patients with higher baseline HbA1c values would have a greater reduction in HbA1c. Increasing insulin sensitivity by testosterone may be explained by various mechanisms: (1) testosterone can upregulate the expression of the insulin receptor, insulin receptor substrate 1, and GLUT4 [28, 29] and (2) testosterone inhibits lipoprotein lipase activity and thus reduces triglyceride flowing into adipocytes. Visceral adipocytes express more androgen receptors than subcutaneous adipocytes [14]; (3) testosterone increases antiinflammatory cytokine IL-10 and decreases proinflammatory cytokines, such as IL-1b, IL-6, and TNF-a. Suppression of the inflammatory state may improve insulin sensitivity [30].

We also found that TRT significantly decreased TG and LDLc levels. Considering their atherosclerogenic effects [31], decreasing TG and LDLc may have promising protective effects on cardiovascular status. Despite this, the role of TRT on HDLc remains controversial. Some studies have shown that TRT may increase HDLc levels [32], while others have not [33]. Our results supported that TRT would not significantly influence HDLc levels. NonRCT studies concluded that testosterone replacement therapy can reduce LDLc and increase HDLc [34]. These results were not fully shown in our research, because it is a nonRCT study, and improvement in dyslipidemia would be achieved after a long period of TRT.

Furthermore, our analysis indicated that TRT resulted in a remarkable reduction in weight, BMI, and waist circumference, reflecting that testosterone therapy would improve abdominal (central) adiposity. On average, TRT leads to a weight loss of 3.91 kg, equal to 4-5% of body weight. This weight loss could partially explain the beneficial effect of TRT on glucose metabolism [32]. Long-term TRT (8 years) can reduce body weight by about 10% in patients with hypogonadism and prediabetes and can successfully prevent the progression to T2DM [35]. The lower reduction of body weight in our meta-analysis, 4-5% as opposed to 10%, is possibly due to the short duration of TRT (3–24 months). Longer treatment periods could result in lower body weights and may thus bring a further reduction in cardiovascular risks. A low level of testosterone is associated with obesity. Obesity may reduce testosterone levels by conversion of testosterone to estrogen in adipose tissue. On the other hand, testosterone deficiency may slow down the metabolism of triglycerides and increase the accumulation of adipose [36, 37]. The supplementation of testosterone leads to weight loss by increasing metabolic function and energy utilization [36, 38, 39].

TRT may partially improve hypogonadism symptoms. The reduction of the AMS score (denoting improvement in aging-related symptoms) in this meta-analysis is consistent with the concept that TRT may improve the androgen deficiency associated with the symptoms of aging [9]. To our disappointment, TRT did not significantly improve the IIEF-5 score (erectile function) in this meta-analysis. Of all 5 studies included, only one by Gianatti showed a negative effect on erectile function resulting from TRT, which influenced the final result. On the one hand, multiple factors are involved in erectile dysfunction [40]. It is possible that neurological and microvascular complications associated with aging and diabetes, rather than low testosterone levels, may be the dominant pathology for erectile dysfunction [12]. These patients would have poor response to TRT. Too short period of observation in RCT studies may be another reason for no improvement in the IIEF-5 score. Therefore, the effect of TRT on erectile function is still uncertain, and long-term RCT studies are needed.

No significant change was observed in systolic and diastolic blood pressure after TRT [10]. One RCT revealed that TRT over a long period (60 months) might lower blood pressure. A recent nonRCT study has confirmed that long-term TRT can result in a significant improvement in arterial stiffness and blood pressure control [41]. Unfortunately, due to few randomized controlled trials, the comprehensive effect of TRT on the vascular wall was not evaluated in our meta-analysis.

The controversy of TRT on CVD is still existing [42]. In recent years, many evidence showed that normalized testosterone is a protective factor for cardiovascular disease [34]. It is found that TRT can reduce the incidence of CVD by improving glycemic and lipid metabolism. A study, lasting for 8 years and focusing on testosterone treatment on CVD, found that TRT reduced not only the incidence of CVD but also all-cause mortality and CVD-induced mortality [41].

TRT can consistently increase hemoglobin and hematocrit, as indicated by our study. Bone marrow in the elderly is more sensitive to testosterone than that in younger men [1]. It has been shown that, during 6∼12 months of TRT, hemoglobin increases and then remains within the normal range [43].

Some studies have brought forth concerns regarding TRT-related prostate hyperplasia and cancer [44]. Our meta-analysis revealed that the physiological dosage of testosterone does not result in an increase in the PSA biomarker. Guidelines were developed to assess the risk of prostate cancer before or during TRT [44, 45]. However, at least three studies found that TRT may reduce the incidence of prostate cancer (1.08% in the TRT group vs. 7.35–9.6% in the control group) [34, 46], possibly by reducing the risk factors, such as antiinflammatory effect and weight loss [47]. The final effects of TRT on prostate cancer should be clarified by future high-quality and large-scale RCTs.

A study found that 97.2% of patients with testosterone deficiency may have multiple comorbidities, including dyslipidemia, hypertension, obesity, type 2 diabetes, and chronic obstructive pulmonary disease (COPD) [39]. TRT can improve all these comorbidities by its beneficial effect on metabolism. TRT may improve COPD by improving respiratory muscle function and by strengthening exercise capacity [48–51]. More studies are needed to further evaluate the effect of TRT on COPD.

Some limitations should be addressed. First, most of the participants in this study have moderate diabetes. Thus, the effect of TRT on patients with poor glycemic control cannot be assessed herein. Second, because most of the patients included are obese, it is, therefore, prudent to extrapolate our conclusion to the nonobese population with T2DM and MetS. Third, the analysis included different routes of administration and dosages of testosterone therapy, which may have influenced the effects of TRT on metabolic factors. Fourth, when PSA and hemoglobin were investigated, only 3 studies were included, yielding results that may potentially be unreliable. Finally, hard indicators of cardiovascular diseases, such as myocardial infarction, heart failure, and cerebral infarction, were not fully evaluated. Studies that include a large sample size and long-term follow-up are needed to demonstrate the favorable effects of TRT on the cardiovascular system.

## 6. Conclusion

TRT can improve multiple cardiovascular risk factors, including blood glucose control, insulin sensitivity, dyslipidemia, and central obesity. Short-term TRT is safe; however, more high-quality RCTs are needed to fully clarify the effects of long-term TRT on cardiovascular disease.

## Figures and Tables

**Table 1:** Characteristics of the randomized clinical studies included in the meta-analysis.

Study, year	Objects	Group	Treatment	Samples	Age	Duration of treatment	BMI	HbA1c
Shigehara, 2017	MetS + free testosterone (FT) ≤11.8 pg/ml	TRT	Testosterone enanthate 250 mg IM every 4 weeks	32	67.0 ± 9.4	12 m	NA	6.5 ± 0.9
		Control	No treatment	33	69.3 ± 9.7		NA	6.3 ± 1.1
Groti, 2018	T2DM + total testosterone (TT) <11 nmol/l and/or free testosterone (FT) level <220 pmol/l	TRT	Testosterone undecanoate (TU) 1000 mg IM every 10 weeks	28	60.2 ± 7.2	12 m	34.0 ± 4.4	8.1 ± 1.0
		Control	Placebo	27			32.6 ± 3.7	7.2 ± 0.8
Khripun, 2018	T2DM + TT <12.1 nmol/L	TRT	1%-transdermal T-gel 50 mg qd + dietary control	40	53.3 ± 5.4	9 m	34.0 ± 1.9	7.8 ± 1.8
		Control	Dietary control	40	54.1 ± 5.6		33.6 ± 2.2	6.7 ± 1.4
Di, 2017	T2DM + TT <12 nmol/L	TRT	TU capsule 80 mg po bid, two weeks later 40 mg bid + hypoglycemic agents	42	44.5 ± 5.7	6 m	26.7 ± 2.4	7.7 ± 2.0
		Control	Hypoglycemic agents	40	45.5 ± 5.2		24.6 ± 2.5	7.6 ± 1.3
Dhindsa, 2017	T2DM + FT <6.5 ng/dL	TRT	TU 250 mg IM every 2 weeks	20	54.6	6 m	39.8 ± 7.8	7.0 ± 1.1
		Control	Placebo	14				
Hackett, 2014	T2DM + FT <225 pmmol/L	TRT	TU 1000 mg IM in the 0th, 6th, and 18th weeks	91	61.2 ± 10.5	7.5 m	33.0 ± 6.1	7.7 ± 1.3
		Control	Placebo	95	62.0 ± 9.3		32.4 ± 5.5	7.5 ± 1.2
Gianatti, 2014	T2DM + TT <12 nmmol/L	TRT	TU 1000 mg IM in the 0th, 6th, and 18th weeks	45	62.0 ± 7.4	10 m	31.5 ± 5.3	6.8 ± 0.9
		Control	Placebo	43			33.4 ± 3.0	7.1 ± 0.6
Jones, 2011	T2D + TT < 11 nmmol/L	TRT	Transdermal testosterone gel 60 mg daily + hypoglycemic agents	108	59.9 ± 9.1	6 m	32.9 ± 6.6	NA
		Control	Placebo + hypoglycemic agents	112	59.9 ± 9.4		31.3 ± 5.4	NA
Aversa, 2010	MetS + TT <3.0 ng/mL	TRT	TU 1000 mg IM every 12 weeks	40	51.6 (49.8–53.4)	12 m	31.0 ± 6.2	6.6 ± 1.3
		Control	Placebo	10	52.8 (50.5–55.0)	12 m	30.2 ± 4.5	5.7 ± 0.5
Kalinchenko, 2010	MetS + TT <12.0 nmol	TRT	TU 1000 mg IM 0th, 6th, and 18th weeks	113	51.6 (49.8–53.4)	7.5 m	35.3 ± 1.8	NA
		Control	Placebo	71	52.8 (50.5–55.0)		34.2 ± 2.1	NA
Kapoor, 2006	T2DM + TT <11.8 nmol/l	TRT	TU 200 mg IM 2 weeks + hypoglycemic agents	12	64.0 ± 1.3	3 m	33.0 ± 0.9	7.3 ± 0.2
		Control	Placebo + hypoglycemic agents	12				
Boyanov, 2003	T2DM + TT <15.1 nmol/l	TRT	TU 120 mg po QD + hypoglycemic agents	24	57.5 ± 4.8	3 m	31.1 ± 4.8	10.4 ± 1.6
		Control	Hypoglycemic agents	24			31.0 ± 4.9	10.3 ± 1.6
Gopal, 2010	T2D + FT <64.8 pg/mL	TRT	TU 200 mg IM every 15 days + conventional treatment	11	44.2 ± 3.3	3 m	23.9 ± 4.5	7.0 ± 2.5
		Control	Placebo + conventional treatment	11				
Francomano, 2014	MetS + TT < 320 ng/dL (11 nmol/L)	TRT	TU 1000 mg IM every 12 weeks	20	58.0 ± 10.0	60 m	31.0 ± 5.0	NA
		Control	No treatment	20	57.0 ± 8.0		31.0 ± 6.0	NA
Yang, 2014	MetS + hypogonadism	TRT	TU 40 mg po bid	60	56.2 ± 5.5	12 m	NA	NA
		Control	No treatment	20			NA	NA
Zhao, 2016	T2D + T <12 mol/L	TRT	TU 20 mg po tid + conventional treatment	35	50.7 ± 5.6	6 m	29.0 ± 4.1	7.2 ± 1.7
		Control	Conventional treatment	30	52.5 ± 3.2		30.3 ± 3.8	7.7 ± 1.2
Wu, 2015	T2D + T <12 mol/L	TRT	TU 250 mg IM QM + conventional treatment	30	45.0∼65.0	6 m	NA	8.7 ± 2.6
		Control	Placebo + conventional treatment	30			NA	8.9 ± 3.0
Heufelder, 2009	T2D + MetS + T <12 mol/L	TRT	Transdermal testosterone gel 50 mg daily + sports + hypoglycemic agents	16	57.3 ± 1.4	12 m	32.1 ± 0.5	7.5 ± 0.1
		Control	Sports + hypoglycemic agents	16	55.9 ± 1.5		32.5 ± 0.5	7.5 ± 0.1

TT, total testosterone; FT, free testosterone; T2D, type 2 diabetes mellitus; TRT: testosterone replacement therapy.

## Data Availability

The data used to support the findings of the study are available from the corresponding author upon request.

## Supplementary Materials

Supplementary Materials Supplementary Figure 1: TRT group has a greater reduction in HbA1c than the control group (subgroup analysis according to TRT period). Supplementary Figure 2: TRT group has a greater reduction in HbA1c than the control group (subgroup analysis according to HbA1c levels before TRT). Supplementary Figure 3: TRT can significantly reduce the FBG level. Supplementary Figure 4: TRT has a greater reduction in FBG (subgroup analysis according to duration of TRT). Supplementary Figure 5: TRT has a significant reduction in FINS. Supplementary Figure 6: TRT can reduce total cholesterol level. Supplementary Figure 7: TRT may reduce the TG level. Supplementary Figure 8: TRT can promote weight reduction. Supplementary Figure 9: TRT reduces waist circumstance. Supplementary Figure 10: TRT increase total testosterone level in serum. Supplementary Figure 12: TRT does not increase PSA. Supplementary Figure 13: TRT increases hemoglobin. Supplementary Figure 14: TRT increases hematocrit. Supplementary Table 1: TRT improves glycemic control. Supplementary Table 2: change in lipid profiles after TRT intervention. Supplementary Table 3: change in body weight and waist circumference after TRT intervention. Supplementary Table 4: changes in safety parameters after TRT intervention.

## References

[1] Bhasin S., Brito J. P., Cunningham G. R. (2018). Testosterone therapy in men with hypogonadism: an endocrine society∗ clinical practice guideline. *The Journal of Clinical Endocrinology & Metabolism*.

[2] Rabijewski M., Papierska L., Zgliczyński W., Piątkiewicz P. (2013). The incidence of hypogonadotropic hypogonadism in type 2 diabetic men in polish population. *Biomed Research International*.

[3] Hall S. A., Esche G. R., Araujo A. B. (2008). Correlates of low testosterone and symptomatic androgen deficiency in a population-based sample. *The Journal of Clinical Endocrinology & Metabolism*.

[4] Dhindsa S., Miller M. G., McWhirter C. L. (2010). Testosterone concentrations in diabetic and nondiabetic obese men. *Diabetes Care*.

[5] Rabijewski M., Papierska L., Piątkiewicz P. (2015). Late-onset hypogonadism among old and middle-aged males with prediabetes in Polish population. *The Aging Male*.

[6] Rendong Z., Lin C., Kemian L., ChuXiaoQiu W. C., Hongping S., Chao L. (2015). Study on sex hormone levels in overweight or obese type 2 diabetic male patients. *International Journal of Endocrinology and Metabolism*.

[7] Tsai E., Boyko E., Leonetti D., Fujimoto W. (2000). Low serum testosterone level as a predictor of increased visceral fat in Japanese-American men. *International Journal of Obesity*.

[8] Basaria S. (2014). Male hypogonadism. *The Lancet*.

[9] Nguyen C. P., Hirsch M. S., Moeny D., Kaul S., Mohamoud M., Joffe H. V. (2015). Testosterone and “age-related hypogonadism”—FDA concerns. *New England Journal of Medicine*.

[10] Francomano D., Lenzi A., Aversa A. (2014). Effects of five-year treatment with testosterone undecanoate on metabolic and hormonal parameters in ageing men with metabolic syndrome. *International Journal of Endocrinology*.

[11] Aversa A., Bruzziches R., Francomano D. (2011). Effects of testosterone undecanoate on cardiovascular risk factors and atherosclerosis in middle-aged men with late-onset hypogonadism and metabolic syndrome: results from a 24-month, randomized, double-blind, placebo-controlled study. *The Journal of Sexual Medicine*.

[12] Gianatti E. J., Dupuis P., Hoermann R., Zajac J. D., Grossmann M. (2014). Effect of testosterone treatment on constitutional and sexual symptoms in men with type 2 diabetes in a randomized, placebo-controlled clinical trial. *The Journal of Clinical Endocrinology & Metabolism*.

[13] Shigehara K., Konaka H., Nohara T. (2018). Effects of testosterone replacement therapy on metabolic syndrome among Japanese hypogonadal men: a subanalysis of a prospective randomised controlled trial (Earth study). *Andrologia*.

[14] Groti K., Žuran I., Antonič B., Foršnarič L., Pfeifer M. (2018). The impact of testosterone replacement therapy on glycemic control, vascular function, and components of the metabolic syndrome in obese hypogonadal men with type 2 diabetes. *The Aging Male*.

[15] Khripun I., Vorobyev S., Belousov I., Kogan M., Zitzmann M. (2018). Influence of testosterone substitution on glycemic control and endothelial markers in men with newly diagnosed functional hypogonadism and type 2 diabetes mellitus: a randomized controlled trial. *Aging Male*.

[16] Di H. J., Fan Y. F., Zhang H. F., Liu K. M., Liu C. (2017). Testosterone undecanoate pills improves insulin resistance in type-2 diabetes men with hypogonadism. *National Journal of Andrology*.

[17] Dhindsa S., Ghanim H., Batra M. (2016). Insulin resistance and inflammation in hypogonadotropic hypogonadism and their reduction after testosterone replacement in men with type 2 diabetes. *Diabetes Care*.

[18] Hackett G., Cole N., Bhartia M. (2014). Testosterone replacement therapy improves metabolic parameters in hypogonadal men with type 2 diabetes but not in men with coexisting depression: the BLAST study. *The Journal of Sexual Medicine*.

[19] Jones T. H., Arver S., Behre H. M. (2011). Testosterone replacement in hypogonadal men with type 2 diabetes and/or metabolic syndrome (the TIMES2 study). *Diabetes Care*.

[20] Kalinchenko S. Y., Tishova Y. A., Mskhalaya G. J., Gooren L. J., Giltay E. J., Saad F. (2010). Effects of testosterone supplementation on markers of the metabolic syndrome and inflammation in hypogonadal men with the metabolic syndrome: the double-blinded placebo-controlled Moscow study. *Clinical Endocrinology*.

[21] Kapoor D., Goodwin E., Channer K. S., Jones T. H. (2006). Testosterone replacement therapy improves insulin resistance, glycaemic control, visceral adiposity and hypercholesterolaemia in hypogonadal men with type 2 diabetes. *European Journal of Endocrinology*.

[22] Boyanov M. A., Boneva Z., Christov V. G. (2003). Testosterone supplementation in men with type 2 diabetes, visceral obesity and partial androgen deficiency. *The Aging Male*.

[23] Yang Z. X. G. J. (2014). Small doses of testosterone supplement therapy on male late-onset hypogonadism with the metabolic syndrome.

[24] Refei Z., Jiang D., Wei Q. K., Xiaoqi G., Hong L. (2017). Effects of testosterone replacement therapy on glycolipid metabolism in type 2 diabetic patients with late onset hypogonadism. *Journal of Practical Diabetology*.

[25] Yan W., Wei W., Al E., Sue S., Liu Z., Chunyan D. (2015). Effect of androgen replacement therapy on late onset hypogonadism in men with diabetes mellitus. *Chinese Journal of Gerontology*.

[26] Heufelder A. E., Saad F., Bunck M. C., Gooren L. (2009). Fifty-two-week treatment with diet and exercise plus transdermal testosterone reverses the metabolic syndrome and improves glycemic control in men with newly diagnosed type 2 diabetes and subnormal plasma testosterone. *Journal of Andrology*.

[27] Gopal R., Bothra N., Acharya S. (2010). Treatment of hypogonadism with testosterone in patients with type 2 diabetes mellitus. *Endocrine Practice*.

[28] Zitzmann M. (2009). Testosterone deficiency, insulin resistance and the metabolic syndrome. *Nature Reviews Endocrinology*.

[29] Polak J., Shimoda L. A., Drager L. F. (2013). Intermittent hypoxia impairs glucose homeostasis in C57BL6/J mice: partial improvement with cessation of the exposure. *Sleep*.

[30] Mohamad N.-V., Wong S. K., Wan Hasan W. N. (2018). The relationship between circulating testosterone and inflammatory cytokines in men. *The Aging Male*.

[31] Corona G., Monami M., Rastrelli G. (2011). Testosterone and metabolic syndrome: a meta‐analysis study. *The Journal of Sexual Medicine*.

[32] Garvey W. T., Mechanick J. I., Brett E. M. (2016). American association of clinical endocrinologists and American college of endocrinology comprehensive clinical practice guidelines for medical care of patients with obesityexecutive summary complete guidelines.

[33] Yassin A. A., El-Sakka A. I., Saad F., Gooren L. J. G. (2008). Lower urinary-tract symptoms and testosterone in elderly men. *World Journal of Urology*.

[34] Saad F., Doros G., Haider K. S., Haider A. (2018). Hypogonadal men with moderate-to-severe lower urinary tract symptoms have a more severe cardiometabolic risk profile and benefit more from testosterone therapy than men with mild lower urinary tract symptoms. *Investigative and Clinical Urology*.

[35] Yassin A., Haider A., Haider K. S. (2019). Testosterone therapy in men with hypogonadism prevents progression from prediabetes to type 2 diabetes: eight-year data from a registry study. *Diabetes Care*.

[36] Traish A. M., Kypreos K. E. (2011). Testosterone and cardiovascular disease: an old idea with modern clinical implications. *Atherosclerosis*.

[37] Kelly D. M., Jones T. H. (2015). Testosterone and obesity. *Obesity Reviews*.

[38] Traish A. M., Guay A., Feeley R., Saad F. (2009). The dark side of testosterone deficiency: I. Metabolic syndrome and erectile dysfunction. *Journal of Andrology*.

[39] Erenpreiss J., Fodina V., Pozarska R., Zubkova K., Dudorova A., Pozarskis A. (2019). Prevalence of testosterone deficiency among aging men with and without morbidities. *The Aging Male*.

[40] Spitzer M., Huang G., Basaria S., Travison T. G., Bhasin S. (2013). Risks and benefits of testosterone therapy in older men. *Nature Reviews Endocrinology*.

[41] Traish A. M., Haider A., Haider K. S., Doros G., Saad F. (2017). Long-term testosterone therapy improves cardiometabolic function and reduces risk of cardiovascular disease in men with hypogonadism. *Journal of Cardiovascular Pharmacology and Therapeutics*.

[42] Weikert C., Pischon T., Weikert S. (2010). Adverse events associated with testosterone administration. *The New England Journal of Medicine*.

[43] Bhasin S., Cunningham G. R., Hayes F. J. (2010). Testosterone therapy in men with androgen deficiency syndromes: an endocrine society clinical practice guideline. *The Journal of Clinical Endocrinology & Metabolism*.

[44] Davidson E., Morgentaler A. (2016). Testosterone therapy and prostate cancer. *Urologic Clinics of North America*.

[45] Morgentaler A., Traish A. M. (2009). Shifting the paradigm of testosterone and prostate cancer: the saturation model and the limits of androgen-dependent growth. *European Urology*.

[46] Wallis C. J. D., Lo K., Lee Y. (2016). Survival and cardiovascular events in men treated with testosterone replacement therapy: an intention-to-treat observational cohort study. *The Lancet Diabetes & Endocrinology*.

[47] Perdana N. R., Mochtar C. A., Umbas R., Hamid A. R. (2016). The risk factors of prostate cancer and its prevention: a literature review. *Acta Medica Indonesiana*.

[48] Svartberg J., Schirmer H., Medbø A., Melbye H., Aasebø U. (2007). Reduced pulmonary function is associated with lower levels of endogenous total and free testosterone. The Tromsø study. *European Journal of Epidemiology*.

[49] Mohan S. S., Knuiman M. W., Divitini M. L. (2015). Higher serum testosterone and dihydrotestosterone, but not oestradiol, are independently associated with favourable indices of lung function in community-dwelling men. *Clinical Endocrinology*.

[50] Creutzberg E. C., Wouters E. F. M., Mostert R., Pluymers R. J., Schols A. M. W. J. (2003). A role for anabolic steroids in the rehabilitation of patients with COPD? A double-blind, placebo-controlled, randomized trial. *Chest*.

[51] Baillargeon J., Urban R. J., Zhang W. (2018). Testosterone replacement therapy and hospitalization rates in men with COPD. *Chronic Respiratory Disease*.

