# Supplementary material for: Metabolic Effects of Testosterone Replacement Therapy in Patients with Type 2 Diabetes Mellitus or Metabolic Syndrome: A Meta-Analysis
Source: Int J Endocrinol. 2020 Sep 30;2020:4732021. doi: 10.1155/2020/4732021 (PMC7545471; doi:10.1155/2020/4732021)

**Supplementary Figure 1** Greater reduction in HbA1c in the TRT group compared to the control group (subgroup analysis according to TRT period)


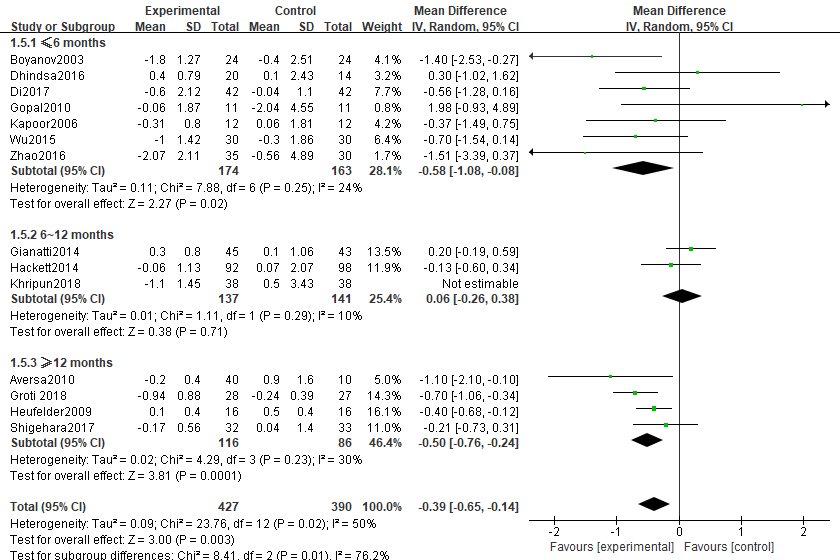


**Supplementary Figure 2** Greater reduction in HbA1c in the TRT group compared to the control group (subgroup analysis according to HbA1c levels before TRT)


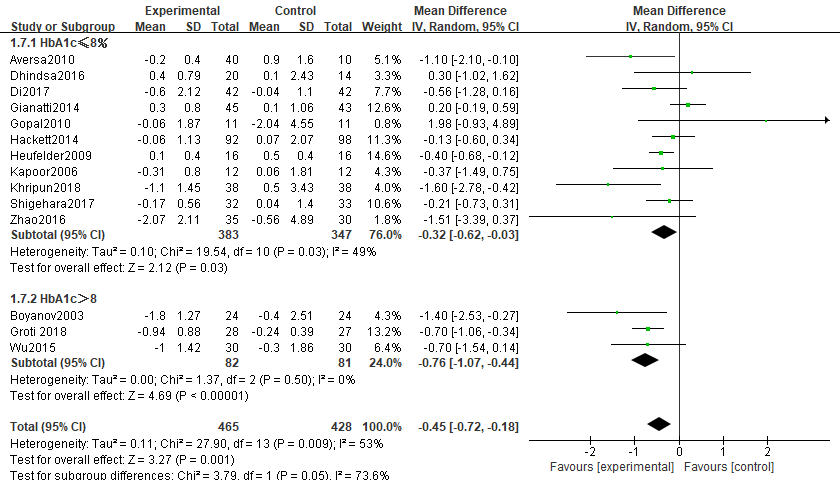


**Supplementary Figure 3** TRT can significantly reduce FBG level
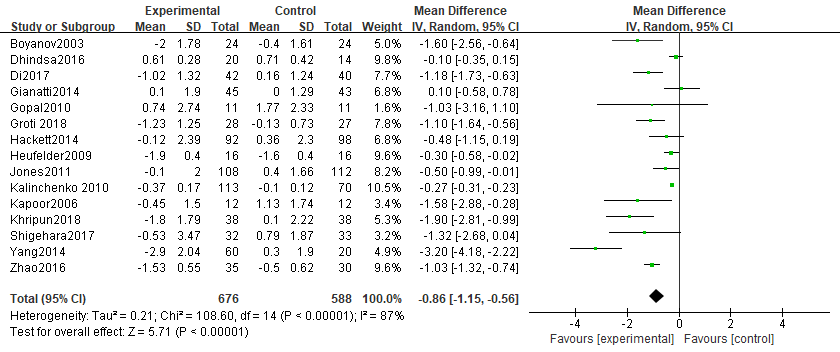


**Supplementary Figure 4** TRT can reduce FBG level (subgroup analysis according to duration of TRT).
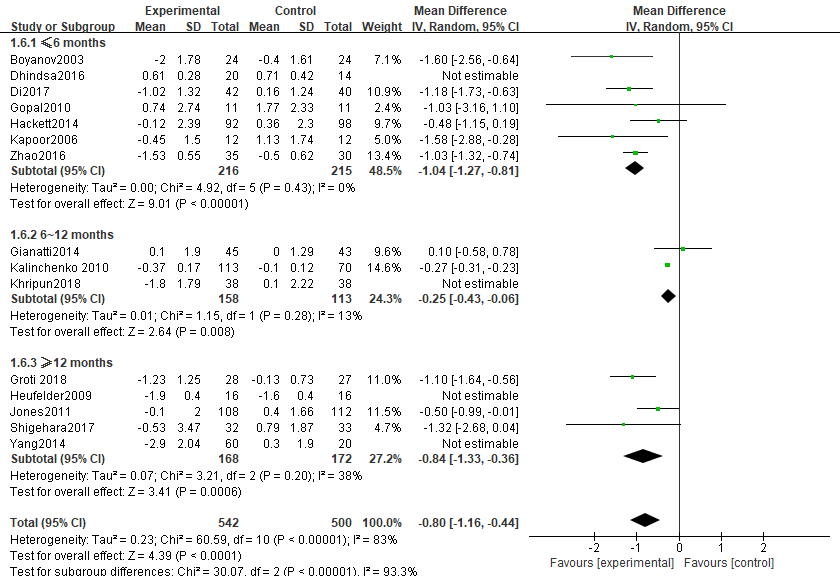


**Supplementary Figure 5** TRT can significantly reduce FINS
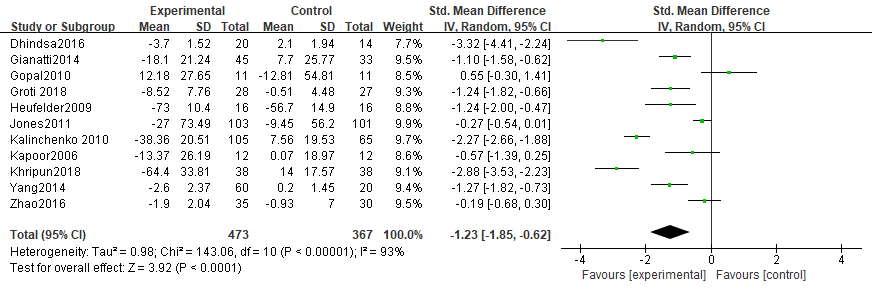


**Supplementary Figure 6** TRT can reduce total cholesterol level


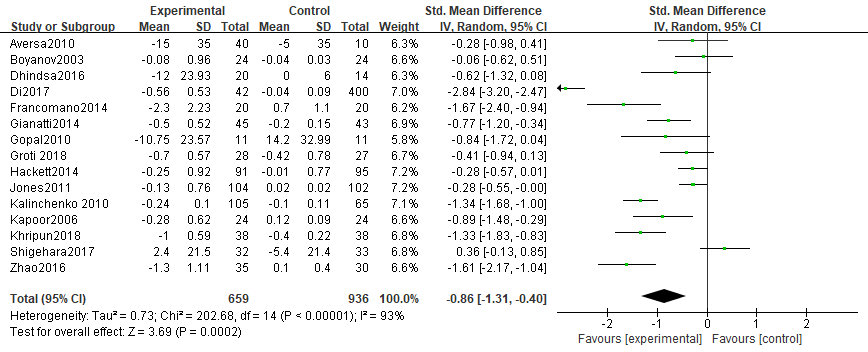


**Supplementary Figure 7** TRT can reduce TG level
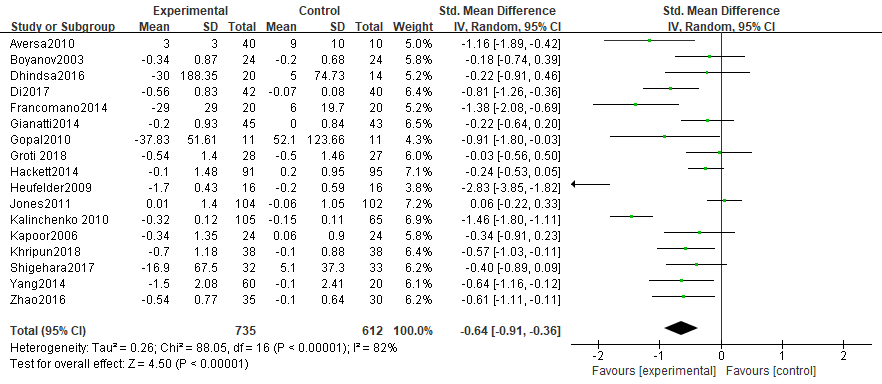


**Supplementary Figure 8** TRT promotes weight reduction


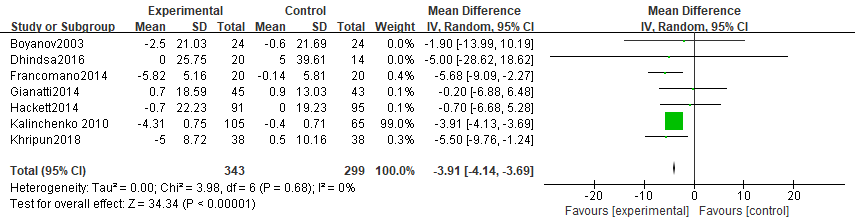


**Supplementary Figure 9** TRT can reduce waist circumstance


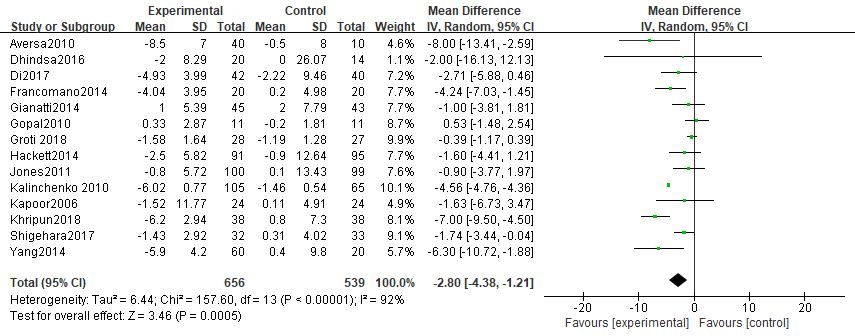


**Supplementary Figure 10** TRT increases total testosterone level in serum
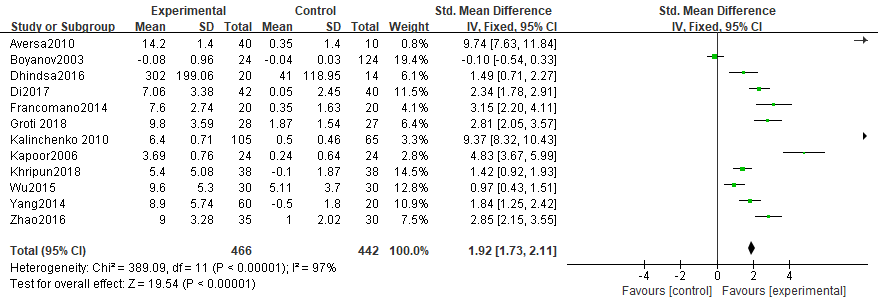


**Supplementary Figure 11** AMS score decreases after TRT


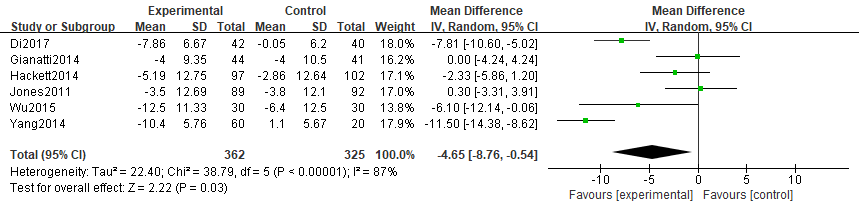


**Supplementary Figure 12** TRT doesn’t increase PSA
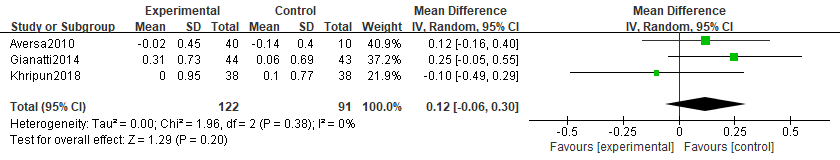


**Supplementary Figure 13** TRT increases hemoglobin


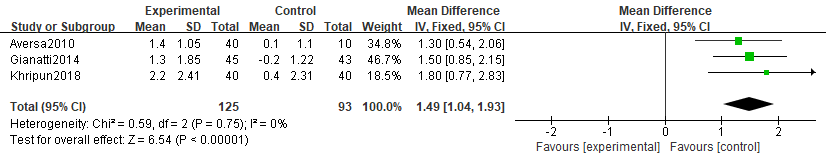


**Supplementary Figure 14** TRT increases hematocrit


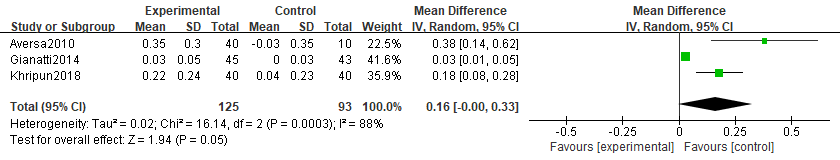

Supplement: Supplementary Materials — Supplementary Figure 1: TRT group has a greater reduction in HbA1c than the control group (subgroup analysis according to TRT period). Supplementary Figure 2: TRT group has a greater reduction in HbA1c than the control group (subgroup analysis according to HbA1c levels before TRT). Supplementary Figure 3: TRT can significantly reduce the FBG level. Supplementary Figure 4: TRT has a greater reduction in FBG (subgroup analysis according to duration of TRT). Supplementary Figure 5: TRT has a significant reduction in FINS. Supplementary Figure 6: TRT can reduce total cholesterol level. Supplementary Figure 7: TRT may reduce the TG level. Supplementary Figure 8: TRT can promote weight reduction. Supplementary Figure 9: TRT reduces waist circumstance. Supplementary Figure 10: TRT increase total testosterone level in serum. Supplementary Figure 12: TRT does not increase PSA. Supplementary Figure 13: TRT increases hemoglobin. Supplementary Figure 14: TRT increases hematocrit. Supplementary Table 1: TRT improves glycemic control. Supplementary Table 2: change in lipid profiles after TRT intervention. Supplementary Table 3: change in body weight and waist circumference after TRT intervention. Supplementary Table 4: changes in safety parameters after TRT intervention. [file 4732021.f1.zip › 4732021.f1/supplementary materials-figures (1).docx]
