# Supplementary material for: Metabolic Effects of Testosterone Replacement Therapy in Patients with Type 2 Diabetes Mellitus or Metabolic Syndrome: A Meta-Analysis
Source: Int J Endocrinol. 2020 Sep 30;2020:4732021. doi: 10.1155/2020/4732021 (PMC7545471; doi:10.1155/2020/4732021)
Supplement: Supplementary Materials — Supplementary Figure 1: TRT group has a greater reduction in HbA1c than the control group (subgroup analysis according to TRT period). Supplementary Figure 2: TRT group has a greater reduction in HbA1c than the control group (subgroup analysis according to HbA1c levels before TRT). Supplementary Figure 3: TRT can significantly reduce the FBG level. Supplementary Figure 4: TRT has a greater reduction in FBG (subgroup analysis according to duration of TRT). Supplementary Figure 5: TRT has a significant reduction in FINS. Supplementary Figure 6: TRT can reduce total cholesterol level. Supplementary Figure 7: TRT may reduce the TG level. Supplementary Figure 8: TRT can promote weight reduction. Supplementary Figure 9: TRT reduces waist circumstance. Supplementary Figure 10: TRT increase total testosterone level in serum. Supplementary Figure 12: TRT does not increase PSA. Supplementary Figure 13: TRT increases hemoglobin. Supplementary Figure 14: TRT increases hematocrit. Supplementary Table 1: TRT improves glycemic control. Supplementary Table 2: change in lipid profiles after TRT intervention. Supplementary Table 3: change in body weight and waist circumference after TRT intervention. Supplementary Table 4: changes in safety parameters after TRT intervention. [file 4732021.f1.zip › 4732021.f1/supplementary materials-tables (1).docx]

**Supplementary Table 1** TRT improves glycemia control

| Author | Year | HbA1c | HBG | FINS | HOMA-IR |
| --- | --- | --- | --- | --- | --- |
| Aversa | 2010 | -0.2±0.4/0.9±1.6 | NA | NA | -2.1±0.35/0.5±0.3 |
| Heufelder | 2009 | 0.1±0.4/0.5±0.4 | -1.9±0.4/-1.6±0.4 | -73±10.4/-56.7±14.9 | -4.2±0.4/-3.4±0.4 |
| Kapoor | 2006 | -0.31±0.8/0.06±1.81 | -0.45±1.5/1.13±1.74 | -13.97±26.19/0.07±18.97 | -0.8±0.87/0.9±3.08 |
| Francomano | 2014 | -2.09±0.51/0.32±0.68 | NA | NA | -2.8±0.38/-1.1±0.33 |
| Di | 2017 | -0.60±2.12/-0.04±1.1 | -1.02±1.32/0.16±1.24 | NA | -1.21±0.64/-0.2±0.38 |
| Gianatti | 2014 | 0.3±0.8/0.1±1.06 | 0.1±1.9/0±1.29 | -18.1±21.24/7.7±26.77 | -0.36±0.53/-0.14±0.51 |
| Hackett | 2014 | -0.06±1.13/0.07±2.07 | -0.12±2.39/0.36±2.3 | -64.4±33.81/14±17.57 | 0.1±1.41/0.27±0.87 |
| Khripun | 2018 | -1.1±1.45/0.5±3.43 | -1.8±1.79/0.1±2.22 | -8.52±7.76/-0.51±4.48 | -3.5±2.15/1.4±1.37 |
| Shigehara | 2017 | -0.17±0.56/0.04±1.4 | -0.53±3.47/0.79±1.87 | NA | -4.64±4.25/-0.52±2.07 |
| Groti | 2018 | -0.94±0.88/-0.24±0.39 | -1.23±1.25/-0.13±0.73 | NA | NA |
| Boyanov | 2003 | -1.8±1.27/-0.4±2.51 | -2±1.78/-0.4±1.61 | NA | NA |
| Gopal | 2010 | -0.06±1.87/-2.04±4.55 | 0.74±2.74/1.77±2.33 | 12.18±27.65/-12.81±54.81 | NA |
| Dhindsa | 2016 | 0.4±0.79/0.1±2.43 | 0.61±0.28/0.71±0.42 | -3.7±1.52/2.1±1.94 | -1.4±1.55/0.3±0.38 |
| Wu | 2015 | -1±1.42/-0.3±1.86 | NA | NA | NA |
| Zhao | 2016 | -2.07±2.11/-0.56±4.89 | -1.53±0.55/-0.5±0.62 | -1.9±2.04/-0.93±7 | -1±1.1/-0.06±1.84 |
| Yang | 2014 | NA | -2.9±2.04/0.3±1.9 | -2.6±2.37/0.2±1.45 | NA |
| Jones | 2011 | NA | -0.1±2/0.4±1.66 | -27±73.49/-9.45±56.2 | NA |
| Kalinchenko | 2010 | NA | -0.37±0.17/-0.1±0.12 | -38.36±20.51/7.56±19.53 | -1.49±0.85/0.2±0.48 |

**Supplementary Table 2** Change in lipid profiles after TRT intervention

|  | Year | Total cholesterol | HDL | LDL | triglycerides |
| --- | --- | --- | --- | --- | --- |
| Aversa | 2010 | -15±35/-5±35 | 3±12/3±6 | NA | 3±3/9±10 |
| Heufelder | 2009 | NA | 0.35±0.4/0.2±0.3 | NA | -1.7±0.43/-0.2±0.59 |
| Kapoor | 2006 | -0.28±0.62/0.12±0.09 | -0.05±0.24/-0.02±0.16 | -0.05±0.78/0.17±0.49 | -0.34±1.35/0.06±0.9 |
| Francomano | 2014 | -2.9±2.51/0.1±1.5 | NA | NA | -41±29/-5±20 |
| Di | 2017 | -0.56±0.53/-0.04±0.09 | 0.04±0.49/-0.06±0.34 | -0.53±0.76/-0.01±0.5 | -0.56±0.83/-0.07±0.08 |
| Gianatti | 2014 | -0.5±0.52/-0.2±0.15 | 0.1±0.39/0.1±0.24 | -0.3±0.69/-0.1±0.42 | -0.2±0.93/0±0.84 |
| Hackett | 2014 | -0.25±0.92/-0.01±0.77 | -0.15±0.67/-0.03±0.26 | -0.1±0.72/-0.05±0.53 | -0.1±1.48/0.2±0.95 |
| Khripun | 2018 | -1±0.59/-0.4±0.22 | 0.11±0.12/0.01±0.08 | -0.55±0.83/-0.01±0.58 | -0.7±1.18/-0.1±0.88 |
| Shigehara | 2017 | 2.4±21.5/-5.4±21.4 | -1.9±7.2/0.32±6.7 | NA | -16.9±67.5/5.1±37.3 |
| Groti | 2018 | -0.7±0.57/-0.42±0.78 | 0.03±0.22/0.02±0.21 | -0.09±0.53/0.25±0.85 | -0.54±1.4/-0.5±1.46 |
| Boyanov | 2003 | -0.08±0.96/-0.04±0.03 | 0.01±0.24/0.02±0.18 | 0.03±1.14/0.08±0.76 | -0.34±0.87/-0.2±0.68 |
| Gopal | 2010 | -10.75±23.57/14.2±32.99 | 7.64±8.07/2.41±9.86 | 22.62±31.62/17.86±31.94 | -37.83±51.61/52.1±123.66 |
| Dhindsa | 2016 | -12±23.93/0±0.6 | -1±9.87/1±9.12 | -10±30.01/-1±16.03 | -30±188.35/5±74.73 |
| Wu | 2015 | NA | NA | NA | NA |
| Zhao | 2016 | -1.3±1.11/0.1±0.4 | NA | NA | -0.54±0.77/-0.1±0.64 |
| Yang | 2014 | NA | 0.1±0.72.0±0.3 | -1.5±0.37/-0.4±0.38 | -1.5±2.08/-0.1±2.41 |
| Jones | 2011 | -0.13±0.76/0.02±0.02 | -0.07±0.37/-0.02±0.22 | -0.13±0.9/-0.04±0.56 | 0.01±1.4/-0.06±1.05 |
| Kalinchenko | 2010 | -0.24±0.1/-0.1±0.11 | -0.076±0.036/0.003±0.068 | --0.39±0.88/-0.16±0.1 | -0.32±0.12/-0.15±0.11 |

**Supplementary Table 3** Change in body weight and waist circumference after TRT intervention

|  | Year | **Weight** | **Waist circumference** | **BMI** |
| --- | --- | --- | --- | --- |
| Aversa | 2010 | NA | -8.5±7/-0.5±8 | -1.2±3/-0.5±6 |
| Heufelder | 2009 | NA | NA | NA |
| Kapoor | 2006 | NA | -1.52±11.77/0.11±4.91 | 0.34±0.9/0.12±0.24 |
| Francomano | 2014 | -15.36±5.25/0.78±6.82 | -9.76±4.02/2.91±5.05 | -2.9±1.63/-1±1.6 |
| Di | 2017 | NA | -4.93±3.99/-2.22±9.46 | -1.56±0.48/-0.24±0.15 |
| Gianatti | 2014 | -1.9±15.71/-1.8±12.96 | 1±5.39/2±7.79 | -0.6±1.08/-0.1±0.2 |
| Hackett | 2014 | -0.7±22.23/0±19.23 | -2.5±5.82/-0.9±12.64 | -0.3±1.22/-0.1±0.2 |
| Khripun | 2018 | -5±8.72/0.5±10.16 | -6.2±2.94/0.8±7.3 | -1.7±0.42/0.3±0.08 |
| Shigehara | 2017 | NA | -1.43±2.92/0.31±4.02 | NA |
| Groti | 2018 | NA | -1.58±1.64/-1.19±1.28 | -0.8±1.17/-0.67±0.87 |
| Boyanov | 2003 | -2.5±21.03/-0.6±21.69 | NA | -1±2.12/-0.13±0.04 |
| Gopal | 2010 | NA | 0.33±2.87/-0.2±1.81 | 0.3±0.57/0.2±0.49 |
| Dhindsa | 2016 | 0±25.75/5±39.61 | -2±8.29/0±26.07 | -0.1±3.37/1.2±0.5 |
| Wu | 2015 | NA | NA | -4.1±3.2/-0.2±1.84 |
| .Zhao | 2016 | NA | NA | NA |
| Yang | 2014 | NA | -5.9±4.2/0.4±9.8 | NA |
| Jones | 2011 | NA | -0.8±5.72/0.1±13.43 | 0.2±1.41/0.16±0.18 |
| Kalinchenko | 2010 | -4.31±-0.75/-0.4±0.71 | -6.02±0.77/-1.46±0.54 | -1.32±0.23/-0.11±0.21 |

**Supplementary Table 4** Changes in safety parameters after TRT intervention

|  | Year | SBP | DBP | PSA | Hematocrit | Hemoglobin |
| --- | --- | --- | --- | --- | --- | --- |
| Aversa | 2010 | 2±10/-2±12 | 3±6/0.5±10 | -0.02±0.45/-0.14±0.4 | 0.35±0.3/-0.03±0.35 | 1.4 ±1.05/0.1 ±1.1 |
| Heufelder | 2009 | -2.5±8.95/-4±5.34 | NA | NA | NA | NA |
| Kapoor | 2006 | NA | 1.14±9.89/-0.48±7.78 | NA | NA | NA |
| Francomano | 2014 | -22.93±10/-3.14±11.25 | -21.6±5.1/-10.4±4.1 | NA | NA | NA |
| Di | 2017 | 0.37±9.76/1.93±6.7 | NA | NA | NA | NA |
| Gianatti | 2014 | 0±13.44/2±9.81 | 1±10/-4±8.56 | NA | 0.03±0.05/ 0±0.03 | 1.3±1.85/-0.2±1.22 |
| Hackett | 2014 | 2.1±17.67/-0.8±9.14 | 0±12.68/-0.2±9.2 | NA | NA | NA |
| Khripun | 2018 | NA | -5±8.72/0.5±10.16 | 0±0.95/0.1±0.77 | 0.22±0.24/0.04±0.23 | 2.2±2.41/0.4±2.31 |
| Shigehara | 2017 | -3.9±12.1/-3.5±15.6 | -0.2±12.2/-1.6±10.6 | 0.31±0.73/0.06±0.69 | NA | NA |
| Groti | 2018 | 4.14±14.99/-2.22±15.71 | 0.64±10.8/-0.16±10.61 | NA | NA | NA |
| Boyanov | 2003 | -2±9.08/-2±5.31 | 2±5.4/4±5.67 | NA | NA | NA |
| Gopal | 2010 | -0.5±8.05/-3.6±6.45 | -2.83±7.26/1±3.16 | NA | NA | NA |
| Dhindsa | 2016 | NA | -5.6±5.13/-4±4.13 | NA | NA | NA |
| Wu | 2015 | NA | NA | NA | NA | NA |
| .Zhao | 2016 | NA | NA | NA | NA | NA |
| Yang | 2014 | -0.3±1.36/0.1±1.21 | -0.1±1.76/0.1±1.25 | NA | NA | NA |
| Jones | 2011 | 0.1±16.17/-1.8±11.16 | 0.3±13.46/-1.3±10.03 | NA | NA | NA |
| Kalinchenko | 2010 | NA | NA | NA | NA | NA |
